# The corrosion mechanism of printed circuit boards affected by haze atmospheric particles

**DOI:** 10.1039/d5ra04326c

**Published:** 2025-08-11

**Authors:** Luntao Wang, Xiong Gao, Hao Zhang, Pan Yi, Yao Tan, Junsheng Wu, Kui Xiao

**Affiliations:** a Institute for Advanced Materials and Technology, University of Science and Technology Beijing Beijing 100083 China xiaokui@ustb.edu.cn; b China Electric Power Research Institute Beijing 100192 China; c China National Electric Apparatus Research Institute Co. Ltd., National Key Laboratory of Environmental Adaptability for Industrial Products Guangzhou 510663 China

## Abstract

As high-density electronic devices face increasing failure rates in polluted environments, understanding particulate-induced corrosion has become crucial. However, the combined effects of particle size, composition, and humidity on printed circuit boards (PCB) corrosion remain poorly understood. This study systematically investigates the corrosion behavior of PCB induced by atmospheric particulate matter under smog conditions. A combination of environmental sampling, compositional analysis, and controlled laboratory corrosion simulations was employed to reveal the synergistic effects of particle size, chemical composition, and humidity on the corrosion process. Particulate matter of different sizes (PM_10_ and PM_2.5_) was collected from northern China, and their morphology, ion content, and interaction with PCB-Cu surfaces were characterized using SEM, EDS, SKPFM, and Raman spectroscopy. The results show that fine particles (1–2 μm) exhibit rapid hygroscopicity and form localized electrolyte films, leading to pitting corrosion *via* oxygen concentration cells. In contrast, coarse particles (5–10 μm) absorb more moisture and induce broader but slower corrosion development. Electrochemical analysis revealed that soluble ions such as NH_4_^+^, SO_4_^2−^, and Cl^−^ play dominant roles in corrosion. Specifically, Cl^−^ disrupts the passive film and accelerates anodic dissolution, while SO_4_^2−^ and high humidity conditions promote the formation of CuO, Cu_2_O, and CuSO_4_·*x*H_2_O. The study confirms that smog particulates facilitate a highly corrosive microenvironment through moisture uptake, ion release, and pollutant gas interaction. These findings offer mechanistic insights into particulate-induced corrosion and provide a theoretical basis for designing corrosion-resistant electronic devices and improving air quality management in industrial regions.

## Abstract 2

As high-density electronic devices face increasing failure rates in polluted environments, understanding particulate-induced corrosion has become crucial.

## Graphical abstract

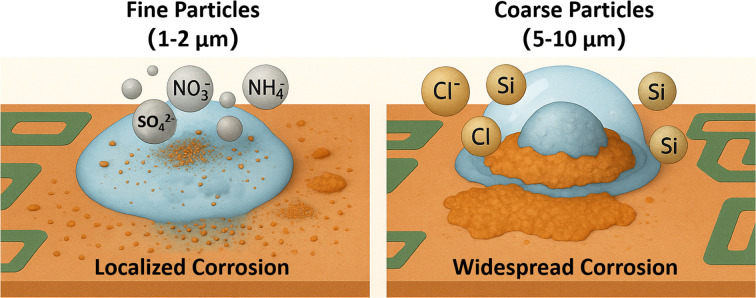

## 1 Introduction

With the rapid advancement of electronic technology, printed circuit boards (PCBs), the core carriers of electronic devices, have been widely applied in high-end fields such as consumer electronics, communication base stations, aerospace, and medical equipment. In recent years, as electronic components evolve toward miniaturization, high density, and multilayer integration, the linewidth and spacing of PCBs have been reduced to the micrometer scale, significantly increasing their sensitivity to environmental factors.^1–3^ In complex atmospheric environments, the reliability of PCBs is highly susceptible to the synergistic effects of humidity, salt spray, corrosive gases, and particulate matter, among which electrochemical corrosion induced by airborne particulates is particularly critical.^4^ Statistics show that approximately 40% to 60% of global electronic equipment failures are directly related to corrosion, and testing data from China indicate that over 60% of corrosion products on thin copper-plated layers contain dust components, highlighting the severity of particulate-induced corrosion.

The frequent occurrence of haze weather has further increased the corrosion risk of PCBs. Haze particles (PM_10_, PM_2.5_) are not only highly concentrated and long-lasting, but their chemical composition is also significantly different from that of ordinary dust. They are rich in soluble ions such as sulfate, nitrate, ammonium salt, and organic pollutants such as polycyclic aromatic hydrocarbons. These components are prone to form high-conductivity electrolyte films under humid conditions, accelerating the anodic dissolution and cathodic reduction reactions of metal conductors, resulting in circuit breakage or short circuit failure of PCB circuits.^5^ For example, sulfate accounts for as much as 20–30% of PM_2.5_ in Beijing in winter, and its reactivity with copper substrates is far higher than that of ordinary dust.^6^ In addition, the high relative humidity (RH > 80%) and temperature fluctuations (20–50 °C) during haze provide ideal conditions for corrosion kinetics, further shortening the service life of PCBs.^7^ Therefore, exploring the corrosion mechanism of haze particles on PCBs has become a key scientific issue in improving the reliability of electronic equipment.

Although studies have shown that atmospheric particulate matter accelerates metal corrosion through the synergistic effect of hygroscopic salts and pollutant gases, there are still deficiencies in the research on the corrosion behavior of PCB-Cu in haze environments. The correlation between particle size and corrosion mechanism is still unclear. The particle size distribution of haze particles is wide (0.1–10 μm), and the deposition pattern, hygroscopicity and chemical activity of particles of different sizes vary significantly. For example, PM2.5 is more likely to adsorb acidic gases (such as SO_2_ and NO_*x*_) due to its large specific surface area, and the local corrosion caused by it may be more severe, but relevant experimental evidence is still lacking.^8–10^ The microscopic characterization of particle corrosion products needs to be further studied. In addition, the global research on the corrosion of haze particles is mostly focused on structural materials such as steel and aluminum alloys,^11–14^ while less attention is paid to electronic materials (such as PCB-Cu), and the relevant conclusions are difficult to be directly extended to the PCB-Cu system. Therefore, it is urgent to combine multi-scale characterization technology methods to systematically analyze the corrosion dynamics and failure mechanism of haze particles on PCB-Cu.

In recent years, the research on the corrosion mechanism of atmospheric particles has made certain progress. Studies have shown that soluble salts (such as (NH_4_)_2_SO_4_, NaNO_3_) are the core driving factors of particle corrosion. Wang *et al.*^15^ systematically explained that in low humidity areas, local corrosion induced by particles is the main cause, while in high humidity areas, uniform corrosion characteristics are shown. It was also verified through indoor simulation experiments that in high humidity environments, particles dissolve to form a continuous liquid film, which accelerates uniform corrosion; in low humidity environments, the liquid film is discontinuous and corrosion is concentrated around the particles, revealing the dynamic interaction between humidity and particles. Xiao *et al.*^16^ studied the corrosion effect of the special dry and hot desert environment (large temperature difference between day and night and high salt dust content) in Turpan, China on PCB-Cu. They found that salt dust not only reduces the critical humidity by absorbing moisture, but also interacts with the temperature difference between day and night to form local corrosion hotspots. They established a corrosion kinetic model of the dynamic interaction between salt dust and temperature difference. Lobnig *et al.*^17^ revealed that submicrometer ammonium sulfate particles can significantly alter copper corrosion behavior at 298 K and 93% relative humidity. As the surface concentration increases from 1 μg cm^−2^ to 10 mg cm^−2^, the formation of basic copper sulfates slows, and incomplete conversion occurs, leaving residual corrosion products on the surface. Although the above results have promoted the development of particle corrosion theory, the experimental data for the PCB-Cu system is still insufficient, especially the corrosion behavior under the multi-component synergistic effect of haze particles has not been systematically revealed.

This study focuses on smog particulate matter in northern China and addresses key knowledge gaps in the corrosion behavior of PCB-Cu exposed to haze environments. Although previous studies have emphasized the role of individual factors such as humidity or specific salts, the synergistic effects of real-world haze particles, characterized by multicomponent chemical compositions and variable particle sizes, on PCB corrosion remain poorly understood. In particular, the relationship between particle size (PM_10_ and PM_2.5_), chemical composition, and corrosion dynamics has not been quantitatively clarified. To bridge this gap, we systematically investigate the deposition characteristics of haze particles of different sizes, their influence on the surface wettability of PCB substrates, and the quantitative correlation between particulate chemical constituents and corrosion rate through controlled indoor simulations. Multiscale characterization techniques including SEM, EDS, localized electrochemical testing, and Raman spectroscopy were employed to elucidate the localized corrosion mechanisms induced by complex particulate matter. The findings offer new perspectives for environmental corrosion research of electronic components and support the development of more robust atmospheric pollution mitigation measures in industrial regions.

## 2 Materials and methods

### 2.1 Experimental materials

**Fig. 1:**
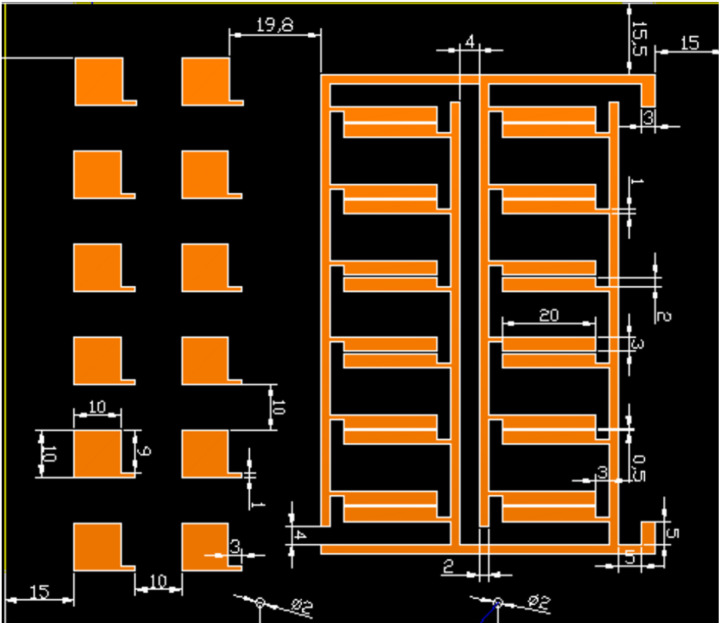
PCB circuit design.

PCB-Cu specimens were used as the experimental material, with the basic parameters summarized in Table 1 and the copper-coated layout shown in Fig. 1. The design includes both discrete copper pads and fine-line copper traces, representing typical structures found in high-density circuit boards. These copper-coated regions served as the primary test areas for corrosion evaluation. Prior to testing, the specimens were ultrasonically cleaned with deionized water and anhydrous ethanol, then air-dried at room temperature.

**Table 1:** PCB-Cu processing parameters

	Plate	Thickness	Copper thickness	Surface treatment	Protective layer thickness
PCB-Cu	FR-4	0.8 mm	25–30 μm	Bare copper without treatment	0 μm

Atmospheric particulate matter used in the corrosion simulation was collected during winter haze events in an industrial–urban transition zone in Beijing, China, where ambient pollution levels are representative of realistic service conditions for electronic devices.

### 2.2 Indoor simulated accelerated experiment

Based on the compositional and morphological analysis of particulate matter in the northern China area, an indoor simulated particulate corrosion test method was designed. Activated carbon powder, known for its porous adsorption properties, was used as a carrier for dissolved ions.

First, a salt solution was prepared by mixing 0.1 g of NH_4_Cl, 0.1 g of (NH_4_)_2_SO_4_, 10 mL of deionized water, 0.02 g of activated carbon powder with a particle size of 2.5 μm, and 90 mL of anhydrous ethanol. The salt solution and activated carbon powder (simulating inert atmospheric particulates, particle size range: 1–10 μm) were then mixed at a mass ratio of 1 : 1 and stirred magnetically for 30 minutes to ensure uniform adsorption of salts onto the activated carbon.

This mixture was diluted with anhydrous ethanol at a volume ratio of 1 : 9 and ultrasonically dispersed for 10 minutes to form a homogeneous suspension. A micropipette was used to drop the suspension onto the surface of PCB-Cu specimens at a controlled volume of 5 μL cm^−2^ to simulate real-world particulate deposition density. The specimens were dried in a vacuum oven for 2 hours (temperature: 25 °C, vacuum: 10 Pa) to ensure that the salts and activated carbon particles adhered firmly to the surface.

For each test condition, at least three replicate PCB-Cu specimens were prepared and analyzed to ensure reproducibility. The PCB-Cu specimens were then placed in a temperature and humidity controlled chamber set at 40 °C and 60% relative humidity. Samples were retrieved at time intervals: 1 hour, 8 hours, and 24 hours. After each sampling, SEM, and other morphological and compositional analyses were conducted immediately. The results shown in this study are representative of repeated measurements, which demonstrated good consistency across all replicates and minimal data variation.

### 2.3 Corrosion characterization methods

To characterize corrosion morphology and product formation over time, a suite of surface analysis techniques was employed. Confocal laser scanning microscopy (CLSM, KEYENCE VK-X) was used to monitor the evolution of corrosion features around deposited particles, enabling visualization of electrolyte film spread and corrosion product distribution.

Scanning electron microscopy (SEM, FEI Quanta 250) operated at an accelerating voltage of 10 kV was used to observe surface morphology. Elemental composition was analyzed using an integrated energy-dispersive X-ray spectroscopy (EDS, EDAX Octane Elect Plus) system, with both spot analysis and elemental mapping performed to determine the distribution of key elements (C, O, S, Cl) related to salt migration and localized corrosion.

Surface Volta potential mapping was conducted using a M370 Scanning Kelvin Probe (SKP, KP Technology, UK). Measurements were performed over a 400 μm × 400 μm area with a step size of 20 μm. A tungsten filament probe was maintained at a fixed distance of 100 ± 3 μm, under ambient conditions (∼25 °C, 45% relative humidity), to identify electrochemical activity on the surface.

Raman spectroscopy was performed using a JY-HR800 micro-Raman spectrometer (HORIBA Jobin Yvon, France) with a 532 nm Torus single-frequency continuous-wave laser and a laser spot diameter of 2–3 μm. A standard laser power of 1 mW was used for general measurements, while a reduced power of 0.1 mW was applied in air to prevent thermal damage to sensitive corrosion products.

## 3 Results and discussion

### 3.1 Analysis of atmospheric particulate matter

**Fig. 2:**
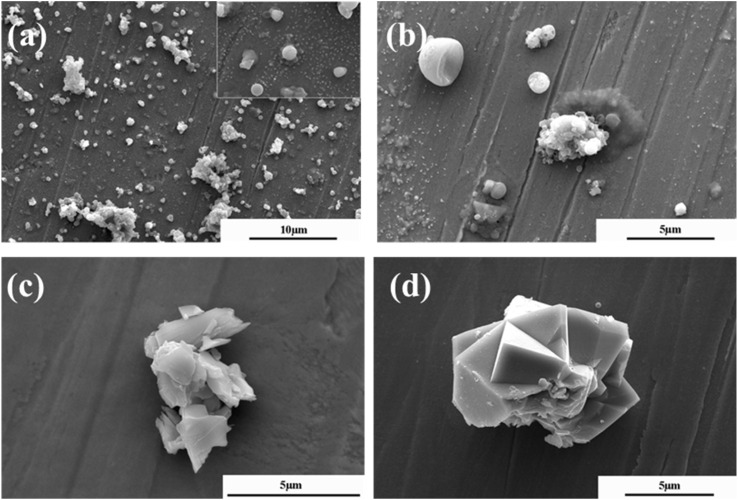
SEM morphology of atmospheric particles of different sizes in northern China area: (a) 1 μm; (b) 2 μm; (c) 5 μm; (d) 10 μm.

The morphology of atmospheric particulate matter during haze weather in northern China area exhibits significant particle size dependence. As shown in Fig. 2, there are obvious morphological differences between particles in different sizes. Particles with smaller diameters (1 μm and 2 μm) have relatively regular morphologies, smooth surfaces, and are generally close to spherical in shape. In contrast, particles with larger diameters (5 μm and 10 μm) have relatively irregular shapes, rough surfaces, and contain many pores. This rough and porous surface morphology is similar to that of activated carbon, and thus they tend to exhibit relatively better physical hygroscopic properties. Coarse particles aggravate visibility degradation through the following pathways. Hygroscopic expansion increases the optical cross-sectional area of particles, promotes secondary aerosol liquid phase reaction to generate sulfate/nitrate (such as NH_4_NO_3_ and (NH_4_)_2_SO_4_), and enhances the light scattering effect as Mie scattering efficiency is increased by about 2 to 3 times.^27^

EDS analysis of atmospheric particulate matter (Table 2) have shown that the elemental compositions of particles with different sizes are correlated with their pollution sources. Fine particles (1 μm and 2 μm) show typical secondary aerosol elemental composition characteristics, with nitrogen and sulfur enrichment being particularly prominent. The mass fractions of N and S in 1 μm particles are 12.54% and 9.93%, respectively, while those in 2 μm particles rise to 17.18% and 8.12%, both significantly higher than the corresponding element contents in coarse particles (5 μm and 10 μm). In addition, the proportion of N and S in the fine particle phase exceeds that of other major elements (Na, Mg, Cl, K, Si, *etc.*), indicating that its formation process is mainly controlled by the secondary aerosol generation mechanism, that is SO_2_ and NO_*x*_ in the atmosphere are converted into sulfate and nitrate under photochemical oxidation.^18^ This process is closely related to coal burning emissions in winter and motor vehicle exhaust emissions throughout the year. Coarse particles show Cl/Si differentiation characteristics, with the chlorine content in 10 μm particles as high as 7.46%, which may come from chloride emissions from surrounding industrial areas. At the same time, the content of crustal elements such as silicon and magnesium increase significantly with increasing particle size, with Si accounting for 21.36% in 10 μm particles, reflecting the important contribution of dust transport on the Mongolian Plateau to coarse particles in spring.^19^

**Table 2:** EDS results of atmospheric particles with different particle sizes (wt%)

	C	N	O	Na	Mg	S	Cl	K	Si
1 μm	33.14	12.54	29.76	4.01	1.05	9.93	2.69	1.36	2.24
2 μm	19.66	17.18	34.43	2.65	0.84	8.12	0.88	1.53	5.12
5 μm	26.90	5.34	40.65	—	6.17	3.40	—	—	9.65
10 μm	36.91	5.60	24.04	—	2.88	1.75	7.46	—	21.36

The most significant contribution of atmospheric particulate matter to corrosion lies in its content of water soluble salts, which exhibit strong hygroscopicity and can form electrolyte layers under humid conditions, thereby facilitating electrochemical corrosion processes.^20^ To investigate this effect, particulate matter of different aerodynamic diameters was collected and analyzed using ion chromatography to determine the concentration of soluble ions. The results are shown in Table 3. Ammonium was the dominant cation, reaching 128.4 mg g^−1^ in 2 μm particles, and readily combines with nitrate and sulfate to form highly hygroscopic salts. These salts exhibit deliquescence at relatively low relative humidity, approximately 62% for ammonium nitrate and 78% for ammonium chloride under ambient conditions.^21^ Their presence lowers the humidity threshold for water uptake and electrolyte layer formation, thereby promoting atmospheric moisture absorption and accelerating corrosion processes.

**Table 3:** Ion chromatography test results of atmospheric particles of different particle sizes

Ion (mg g^−1^)	NH_4_^+^	Mg^2+^	Cl^−^	NO_3_^−^	SO_4_^2−^	Total
1 μm	91.4	7.6	23.1	65.1	75.3	262.5
2 μm	128.4	6.1	7.6	55.1	61.8	259.0
5 μm	51.6	45.0	3.2	53	25.6	178.4
10 μm	62.4	21.1	64.3	21.4	13.3	182.5

Cl^−^ and NO_3_^−^ were primarily enriched in fine particles (1 μm), with concentrations of 23.1 mg g^−1^ and 65.1 mg g^−1^, respectively. Their high solubility facilitates droplet nucleation and prolongs haze persistence.^22^ Sulfate was most concentrated in fine particles as well, peaking at 75.3 mg g^−1^ in 1 μm samples, which is nearly 5.7 times higher than that in coarse 10 μm particles (13.3 mg g^−1^). This enrichment is likely related to heterogeneous oxidation on dust surfaces, such as Fe^3+^ catalyzed oxidation of SO_2_.^23^

It is noteworthy that ion chromatography indicated Cl^−^ was more concentrated in coarse particles, with a content of 64.3 mg g^−1^ in 10 μm particles, whereas EDS in Table 2 revealed a similar Cl^−^ enrichment trend. This apparent discrepancy can be explained by the chemical speciation of chlorine. In coarse particles, Cl^−^ is likely present in sparingly soluble forms such as FeCl_3_ or AlCl_3_, which inhibit ionization.^34^ In contrast, Cl^−^ in fine particles exists in a more reactive ionic state and is prone to participating in heterogeneous reactions, upon reaction with NO_*x*_, thereby aggravating secondary pollution during haze episodes.^24^

The total content of water-soluble salts in particulate matter ranged from 178.4 to 262.5 mg g^−1^, accounting for approximately 18–26% of particle mass, contributing to haze-related corrosion through several mechanisms. Hygroscopic salts lower the activation threshold for cloud condensation nuclei, enhancing aerosol–cloud interactions. The resulting electrolyte solutions accelerate liquid-phase oxidation processes, such as the conversion of SO_2_ to H_2_SO_4_, which facilitates the onset of secondary pollution.^25^ Furthermore, the porous structure and internal voids of atmospheric particles significantly enhance multiple light scattering within the particle body, thereby increasing the aerosol extinction coefficient at 550 nm.^26^

The above analysis reveals the physicochemical characteristics of particulate matter and its environmental effects during haze episodes in northern China area. Fine particles predominantly drive chemical formation processes, particularly the generation of secondary aerosols, while coarse particles are more associated with physical hygroscopicity and dust transport. The size-dependent distribution of chlorine species provides a new perspective for pollution source identification. The coupling of hygroscopic growth, chemical reactions, and optical effects across multiple scales is identified as a key driving force behind the persistence of haze.

### 3.2 Corrosion morphology analysis

Particulate matter with diameters of 1, 2, 5, and 10 μm was deposited onto PCB-Cu substrates and subjected to an indoor corrosion simulation under controlled temperature (40 °C) and relative humidity (60%). Samples were retrieved after 1 h and 8 h of exposure, and their surface morphologies were examined using a laser confocal microscope.

**Fig. 3:**
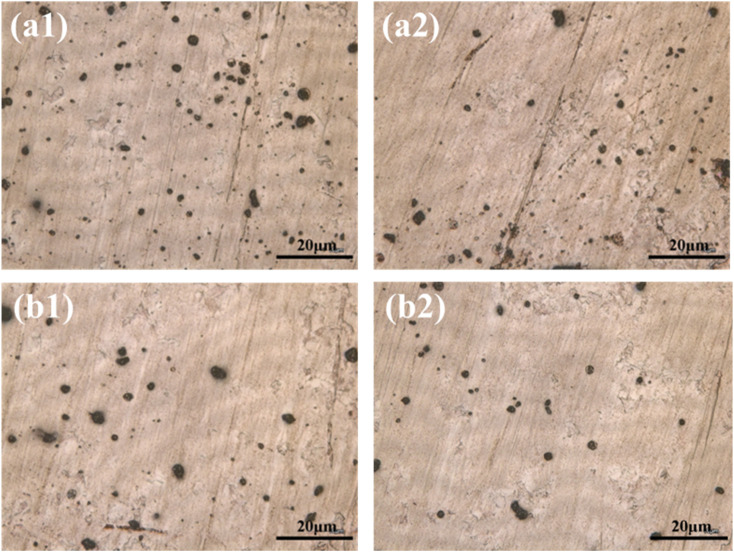
Surface morphologies of PCB-Cu exposed to (a) 1 μm and (b) 2 μm particles after 1 h (a1 and b1) and 8 h (a2 and b2) of indoor simulated corrosion.

As shown in Fig. 3, small particles (1 μm and 2 μm) rapidly formed distinct moisture rings around them within the first hour of exposure. As the experiment progressed, both the number of particles exhibiting such rings and the size of the rings increased. However, the overall ring size remained relatively small, typically not exceeding half the diameter of the particles. This indicates that while fine particles possess strong hygroscopicity, the extent of moisture adsorption is limited by their size, resulting in a relatively localized effect.

**Fig. 4:**
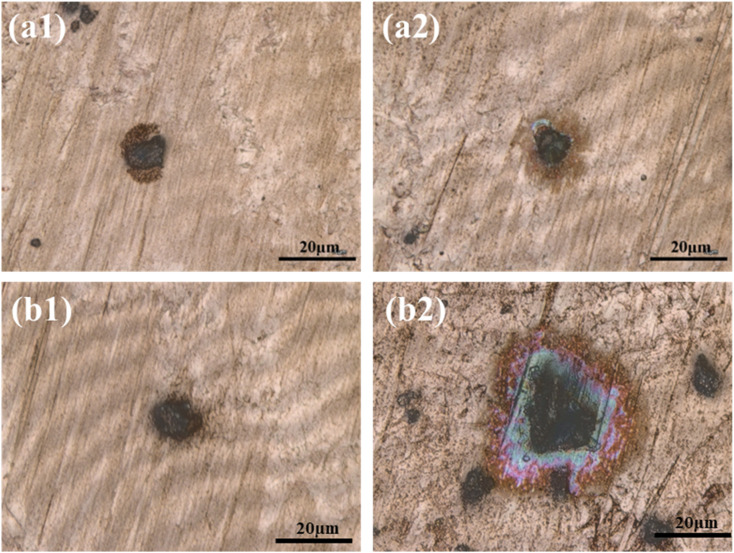
Surface morphologies of PCB-Cu exposed to (a) 5 μm and (b) 10 μm particles after 1 h (a1 and b1) and 8 h (a2 and b2) of indoor simulated corrosion.

As shown in Fig. 4(a), the corrosion behavior around medium-sized particles (5 μm) developed more slowly during the initial stage of the experiment compared to smaller particles. However, as time progressed, a clearly defined corrosion zone began to emerge and continued to expand steadily. Eventually, a relatively large corroded area formed, with a diameter reaching up to three times that of the original particle. This may be attributed to the particle's ability to adsorb and retain larger micro-droplets. Due to gravitational effects, the surface tension becomes insufficient to maintain the droplet's shape, causing it to spread over the surface and generate a wider corrosion region.

In Fig. 4(b), the corrosion process associated with larger particles (10 μm) follows a trend similar to that of the 5 μm particles but develops even more slowly at the early stage. Initially, droplet formation around the particle is delayed. Once a droplet forms, however, the corrosion area expands rapidly and extensively. This behavior may be explained by the requirement for a greater amount of adsorbed moisture to form a stable droplet around the larger particle. Once formed, the droplet quickly deforms and collapses under gravity, spreading into a thin liquid film around the particle and resulting in rapid corrosion spread.

Particles with different sizes exhibit distinct corrosion behaviors in indoor accelerated experiments. Fine particles show strong hygroscopicity but have a limited area of influence. Medium-sized particles develop corrosion zones in a steady and sustained manner, while large particles, once droplet formation occurs, lead to rapid and extensive corrosion spread. These differences provide important insights into the underlying corrosion mechanisms of particulate matter under hot and humid conditions.

### 3.3 Corrosion products analysis

**Fig. 5:**
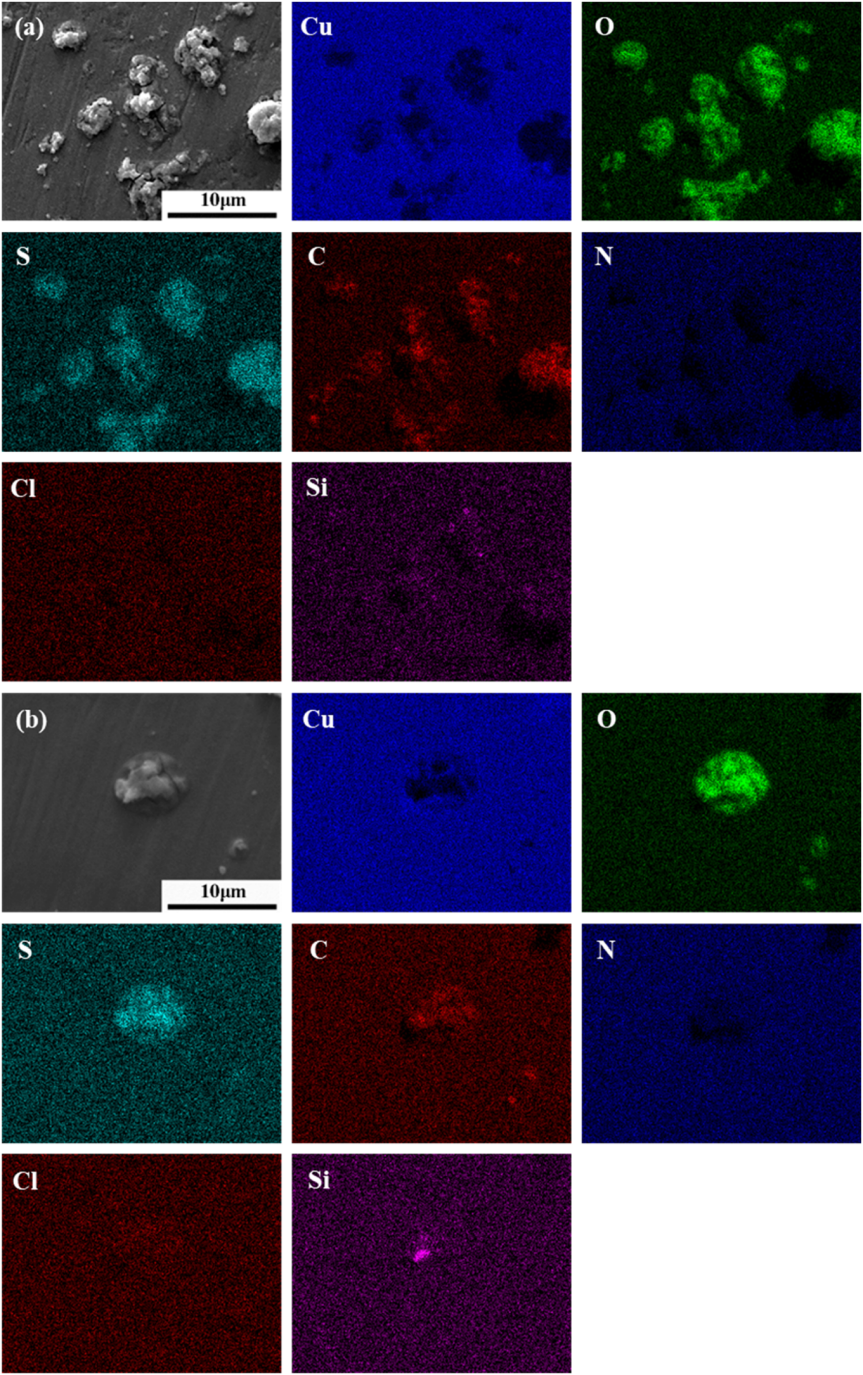
SEM and EDS analysis of PCB-Cu after corrosion induced by (a) 1 μm and (b) 2 μm particles.

As shown in Fig. 5(a), the microscopic morphology of corrosion induced by 1 μm particles reveals the formation of uneven corrosion products within a 1–2 μm region surrounding the particles, with some areas exhibiting cracks. EDS elemental mapping indicates that oxygen and sulfur are co-located with the particles and corrosion products, suggesting a high sulfur content involved in the corrosion process. In contrast, carbon is distributed over a smaller area compared to sulfur and oxygen, indicating that it originates from the particles but does not participate in corrosion product formation. Nitrogen and chlorine are more widely distributed in certain areas near the particles. However, their absence in some regions appears to correlate with particle geometry rather than actual elemental deficiency. Silicon is detected only in a small portion of the particles, likely not involved in corrosion, but possibly serving as a nucleation core for the adsorption of other elements.

As shown in Fig. 5(b), the corrosion morphology and elemental distribution associated with 2 μm particles are similar to those observed for the 1 μm particles, with the formation of rough, uneven corrosion products. The EDS elemental mapping exhibits similar patterns, indicating that the corrosion processes are comparable.

**Fig. 6:**
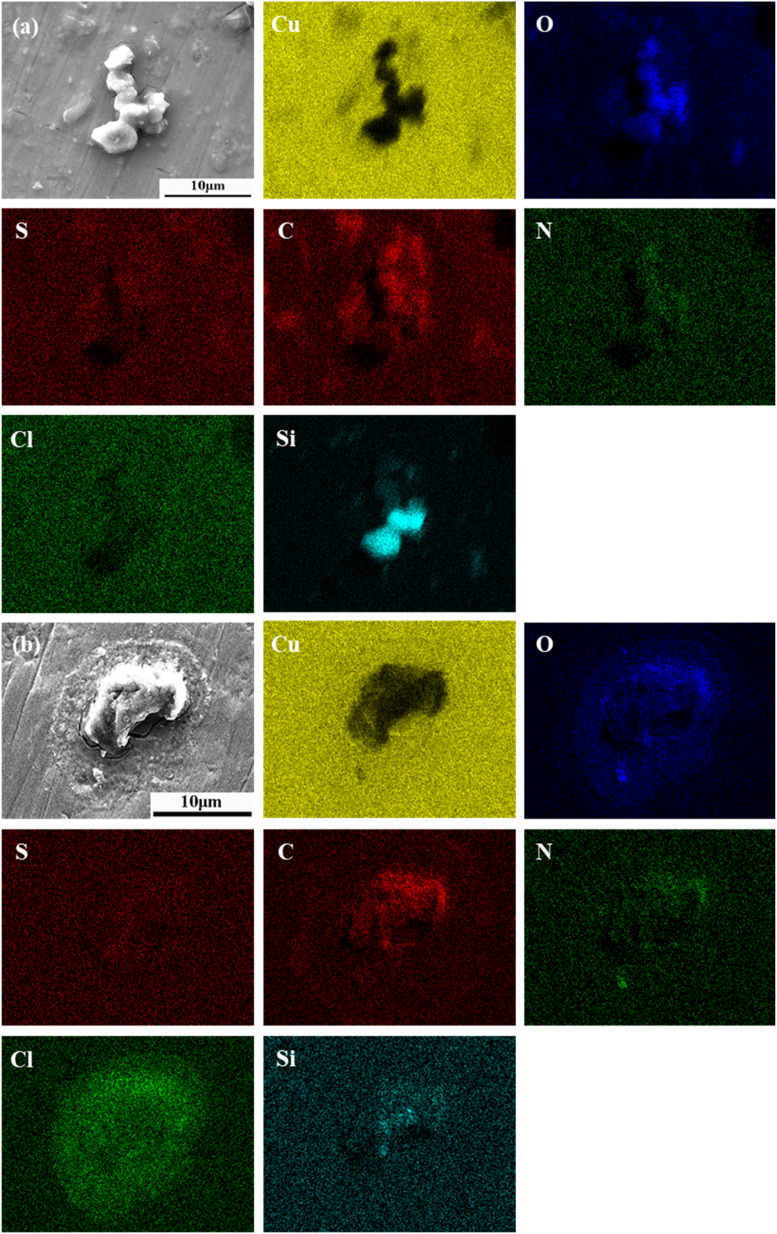
SEM and EDS analysis of PCB-Cu after corrosion induced by (a) 5 μm and (b) 10 μm particles.

As shown in Fig. 6(a), the corrosion morphology around 5 μm particles reveals the presence of cracks in the substrate near the particles. The corrosion product layer appears thin and fractured, but the affected area is relatively large. EDS elemental mapping shows a widespread distribution of oxygen, indicating extensive corrosion in this region. Sulfur and nitrogen are present in low concentrations but co-located with oxygen, suggesting their minor involvement in the corrosion process. Carbon is abundant and generally overlaps with the oxygen distribution, though its intensity varies across different regions. Silicon is unevenly distributed within the particles, suggesting a complex particle formation mechanism.

As shown in Fig. 6(b), corrosion induced by 10 μm particles exhibits a distinct corroded ring centered around the particle. A reduced oxygen signal in the lower central region may be attributed to the particle's morphology impeding electron transfer. EDS analysis shows that oxygen is mainly distributed in the particle and surrounding corroded areas. Nitrogen and silicon are concentrated within the particle itself and are not involved in corrosion product formation. Carbon shows some degree of enrichment in the corroded region. Sulfur does not exhibit noticeable enrichment. Chlorine is abundantly present in both the particle and corrosion products, indicating that chloride ions play a dominant role in the corrosion process.

**Fig. 7:**
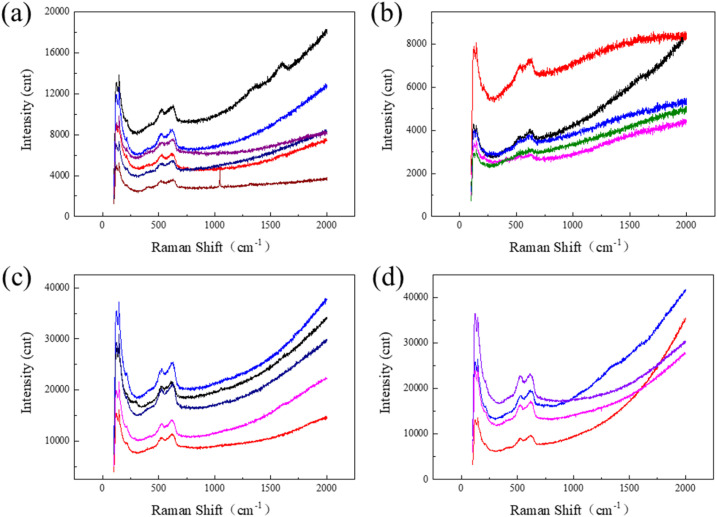
Raman spectra of corrosion products on PCB-Cu surfaces after exposure to particles with different sizes: (a) 1 μm; (b) 2 μm; (c) 5 μm; (d) 10 μm.

Fig. 7 presents the Raman spectroscopy results of PCB-Cu samples after 24 h corrosion induced by particles with different sizes. For fine particles (1 μm and 2 μm), the corrosion products primarily consist of CuO, as indicated by characteristic peaks at 125, 146, 215, 404, 525, and 623 cm^−1^, and Cu(OH)_2_, identified by OH^−^ vibrational peaks at 1360 and 1600 cm^−1^. In some corrosion products associated with 1 μm particles, a distinct peak at 1030 cm^−1^ corresponding to S–O bond vibrations suggest the presence of sulfates, indicating that sulfur participated in the corrosion process. For larger particles (5 μm and 10 μm), CuO was also detected; more importantly, a peak at 290 cm^−1^, corresponding to the stretching vibration of Cu–Cl bonds, indicates the formation of copper chlorides, highlighting the dominant role of chloride in the corrosion mechanism. Although sulfates are not the main components of the corrosion products associated with larger particles, they may still be present to some extent, particularly in regions where sulfur and oxygen co-localize.

Therefore, as the particle size of deposited matter on the PCB-Cu surface increases, the morphology of the corrosion products evolves from uneven and rough structures to thinner, cracked layers, with a broader affected area. Sulfur plays a significant role in the corrosion induced by fine particles, whereas chlorine is more critical in the case of larger particles. The main corrosion products include CuO and Cu(OH)_2_. Copper sulfates are likely to form around fine particles, while copper chlorides dominate in the vicinity of larger particles.

**Fig. 8:**
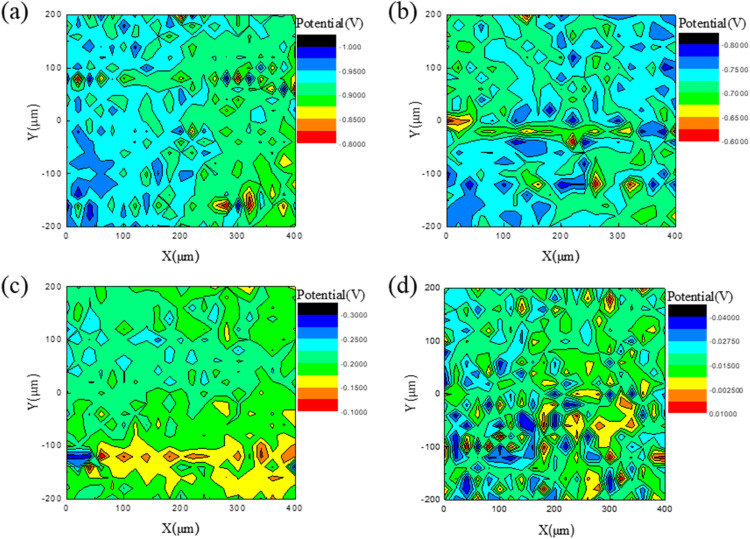
SKP analysis of corrosion potential distribution on PCB-Cu surfaces after exposure to particles with different sizes: (a) 1 μm; (b) 2 μm; (c) 5 μm; (d) 10 μm.

To further investigate the electrochemical influence of particulate matter, localized surface potential mapping was performed using scanning Kelvin probe force microscopy (SKPFM) to assess the corrosion potential differences around deposited particles. The results are shown in Fig. 8.

The corrosion potential distribution associated with fine particles (1 μm and 2 μm) indicates that regions with extreme surface potentials (either highly positive or negative) are limited in size, typically with diameters of 20 μm or less. Potential fluctuations are concentrated around the particles, suggesting a localized influence. Although these fluctuations are confined to small areas, they include regions with significantly low potentials, indicating a strong tendency for localized corrosion. The overall surface potential of samples containing fine particles is relatively low, with Gaussian mean values of −0.9226 V and −0.7201 V for the 1 μm and 2 μm samples, respectively, suggesting that these surfaces are more susceptible to corrosion. Combined with the Raman analysis, corrosion products around the 1 μm particles include not only CuO and Cu(OH)_2_, but also copper sulfates, indicating a complex and highly localized corrosion process.

In contrast, the corrosion potential distribution for larger particles (5 μm and 10 μm) shows that the regions of extreme potential are much larger, reaching up to 50 μm in diameter, with a broader spatial impact. The overall potential of these samples increases with particle size, with Gaussian mean values of −0.1964 V and −0.0169 V for the 5 μm and 10 μm samples, respectively. Although the average potential is higher, low-potential regions are still present and, due to their larger extent, may lead to more widespread corrosion. While large particles may not directly induce intense localized corrosion due to the surrounding high-potential areas, their broad influence increases the risk of uniform corrosion or widespread localized damage. Raman analysis further reveals that, in addition to CuO and Cu(OH)_2_, copper chlorides are present around the 5 μm particles, highlighting the diversity of corrosion products formed (Table 4).

**Table 4:** Average potential fitting results of PCB-Cu surface corrosion of particles with different particle sizes

Particle size	1 μm	2 μm	5 μm	10 μm
Gaussian mean (V)	−0.9226	−0.7201	−0.1964	−0.0169
Standard deviation (V)	0.0299	0.0346	0.02922	0.0106

**Fig. 9:**
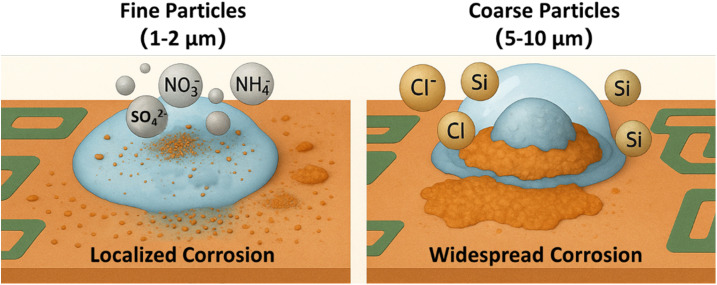
Schematic diagram of particle size and compositions effects on corrosion behavior.

Therefore, particles of different sizes exhibit distinct electrochemical corrosion behaviors. Fine particles primarily induce localized and severe corrosion, characterized by sharp potential fluctuations within a limited spatial range. In contrast, larger particles may lead to more widespread corrosion across the sample surface. Although their overall surface potential is higher, regions with extremely low potential still exist and extend over a broader area. These differences are closely related to particle size effects, the distribution of surface potential, and the composition of the resulting corrosion products (Fig. 9).

### 3.4 Corrosion mechanism analysis

#### 3.4.1 The effect of particle size

The particle size of particulate matter directly affects its deposition state on the surface, its moisture absorption capacity, the characteristics of liquid film expansion, and the spatial distribution of the corrosion area. Fine particles (1–2 μm) have a large specific surface area and are very easy to absorb moisture to form a local electrolyte liquid film, which can quickly induce corrosion even at moderate humidity. In the experiment, it can be observed that these fine particles form a clear wet halo on the copper surface and induce strong local corrosion in a short time. Due to its small particle size, the liquid film is easy to be “coated”, which limits the diffusion of oxygen, forming a low oxygen zone in the center of the particle and a high-oxygen zone at the edge, resulting in a significant “oxygen concentration cell” effect. SKPFM data show that its corrosion potential distribution range is small but fluctuates violently, and the tendency to local corrosion is strong.

In contrast, the hygroscopic behavior of medium coarse particles (5–10 μm) is more significantly affected by morphology and gravity. This type of particle has a strong physical hygroscopic capacity due to its porous and rough surface, but it needs to accumulate more water vapor to form a liquid film. After the liquid film is formed, the droplets tend to collapse and expand under the action of gravity, resulting in a rapid expansion of the corrosion area, which manifests as extended corrosion with a wide range of corrosion and shallow depth. SKPFM shows that its corrosion potential fluctuations are relatively mild, but the low potential area is large, indicating that although it does not induce severe pitting, it is easy to cause wide-area damage modes such as uniform corrosion or under-film corrosion.

#### 3.4.2 The influence of particle compositions

The chemical composition of particulate matter is the core factor that determines its corrosion-inducing ability, especially the type, concentration and physical and chemical form of water-soluble ions, which directly affect the electrochemical path, reaction rate and formation mode of corrosion products. Results have shown that fine particles (1–2 μm) are rich in sulfate (SO_4_^2−^), nitrate (NO_3_^−^) and ammonium ions (NH_4_^+^), which can reach 75.3 mg g^−1^, 65.1 mg g^−1^ and 128.4 mg g^−1^ respectively, showing typical secondary aerosol characteristics. The liquid film formed by these ions after moisture absorption has significant acidity and high conductivity, which reduces the interfacial resistance, increases the charge migration rate, and significantly accelerates the anodic dissolution reaction of copper.^27–29^ This type of acidic salt is prone to ionization and hydration in a humid environment, thus forming a strong local corrosion environment in the particle deposition area. Raman analysis results show that the characteristic peak of S–O bond vibration near 1030 cm^−1^ can be detected in the corrosion products, proving that SO_4_^2−^ participates in the generation of sulfur-containing corrosion products such as CuSO_4_·*x*H_2_O during the corrosion process.^30^ This type of product has a loose crystal structure and high porosity, cannot effectively cover and passivate the copper surface, and is prone to swelling, dissolution or shedding, resulting in protection failure, further exposing the metal surface to corrosive environment.^31,32^

In contrast, the Cl^−^ concentration in coarse particles (5–10 μm) increased significantly, such as 64.3 mg g^−1^ in 10 μm particles, and EDS characterization showed that it was enriched in the corrosion area in a planar manner. This type of chloride ion can not only quickly participate in the electrochemical reaction after absorbing moisture, but also generate corrosion intermediates such as CuCl and CuCl_2_^−^ by complexing copper ions, resulting in continuous dissolution and redeposition of copper, thereby weakening the formation and stability of the passivation film.^33–36^ The Cu–Cl stretching vibration peak at 290 cm^−1^ was detected in the Raman spectrum, further confirming the formation of copper chloride products.

In addition, coarse particles also contain a large number of crustal elements, such as Si (up to 21.36%) and Mg. Most of these components exist in the form of insoluble minerals (such as silicates and carbonates). Although they do not directly participate in the electrochemical reaction themselves, they can form a porous adsorption skeleton on the surface of the particles, acting as a “carrier” for Cl^−^, prolonging its residence time in the surface liquid film and promoting its slow release and migration.^37,38^ As a result, the action of Cl^−^ on the corrosion interface is more continuous and extensive, thus inducing larger-scale subfilm corrosion or uniform corrosion propagation.

## 4 Conclusions

This study systematically investigated the composition and morphology of haze particles in northern China area and their corrosion effects on PCB-Cu. By combining indoor simulation experiments with multi-scale characterization techniques, the corrosion mechanisms of atmospheric particulate matter on electronic components were elucidated.

(1) Particle size significantly influences corrosion behavior. Fine particles (1–2 μm) exhibit high hygroscopicity and readily form localized electrolyte films, leading to intense pitting corrosion. In contrast, medium and coarse particles (5–10 μm) form larger droplets that spread under gravity, resulting in widespread but shallow uniform or under-film corrosion.

(2) Ionic composition determines corrosion product types and mechanisms. Fine particles are rich in SO_4_^2−^, NO_3_^−^, and NH_4_^+^, forming acidic films and generating CuSO_4_·*x*H_2_O products with porous structures. Coarse particles contain high Cl^−^ levels, promoting CuCl formation and undermining passivation, thus causing more extensive corrosion.

(3) Crustal elements (*e.g.*, Si, Mg) in coarse particles, though electrochemically inactive, act as Cl^−^ carriers, enhancing its persistence and corrosivity.

(4) SKPFM results show a strong correlation between potential distribution and corrosion morphology. Fine particles induce sharp, localized potential fluctuations; coarse particles create larger low-potential regions, increasing the risk of widespread corrosion.

Despite the insights gained, this study was conducted under controlled indoor conditions, which may not fully reflect the complex and dynamic nature of real-world atmospheric environments. Future studies should focus on long-term field exposure tests and explore the combined effects of additional environmental stressors such as ultraviolet radiation, temperature fluctuations, and pollutant gases (*e.g.*, SO_2_, NO_*x*_). These efforts will help establish a more comprehensive understanding of particulate-induced corrosion and guide the development of more robust corrosion mitigation strategies for electronic devices in polluted atmospheres.

## Conflicts of interest

The authors declare that they have no known competing financial interests or personal relationships that could have appeared to influence the work reported in this paper.

## Data Availability

The data of this study are available from the corresponding author upon reasonable request.
